# Complete Genome Sequence of *Salmonella enterica* Lytic Bacteriophage LPST10, Isolated in China

**DOI:** 10.1128/genomeA.00427-17

**Published:** 2017-08-31

**Authors:** Xingxing Dong, Chenxi Huang, Mohamed Khairy Morsy, Zhi Li, Yang Zhou, Stephan P. Willias, Hazem Elewa Abdelnabby, Jie Liu, Liping Feng, Xiaohong Wang, Jinquan Li

**Affiliations:** a Key Laboratory of Environment Correlative Dietology, State Key Laboratory of Agricultural Microbiology, College of Food Science and Technology, Huazhong Agricultural University, Wuhan, Hubei, People’s Republic of China; b Department of Food Science, Faculty of Agriculture, Benha University, Qaluobia, Egypt; c Department of Infectious Diseases and Pathology, University of Florida, Gainesville, Florida, USA; d College of Fisheries, Huazhong Agricultural University, Wuhan, Hubei, People’s Republic of China; e College of Medicine, Hebei University of Engineering, Handan, Hebei Province, People’s Republic of China; f Shanghai Laboratory Animal Research Center, Shanghai, People’s Republic of China

## Abstract

Bacteriophage LPST10 was isolated from Wuhan, China. Lytic activity was demonstrated against multiple *Salmonella enterica* serovars, including *Salmonella enterica* serovar Typhimurium strains. This bacteriophage has a 47,657-bp double-stranded DNA genome encoding 87 putative coding sequences.

## GENOME ANNOUNCEMENT

Salmonellosis remains one of the most frequent foodborne zoonoses, constituting a worldwide major public health concern (1). To date, 2,579 *Salmonella* serovars have been identified. Of these, *S. enterica* serovar Typhimurium is one of the most common serovars observed in clinical practice (2). The pathogen-specific antimicrobial activity of lytic phages provides attractive therapeutic potential and has been applied in many commercial products (3, 4).

We isolated and characterized a lytic phage, LPST10, from the environment using *S*. Typhimurium ATCC 14028 as the host. Morphological analysis by transmission electron microscopy revealed that phage LPST10 belongs to the family *Siphoviridae* (5). It has an isometric head of about 83 nm in diameter and a long noncontractile tail of 145 nm. This phage exhibits a broad host range capable of lysing *Salmonella* species across multiple serovars. Phage LPST10 has been successfully applied in milk, sausage, and lettuce as a biocontrol agent against *Salmonella* species, and a patent application has been submitted in China (J. Li, 29 October 2016, China Patent Office). The complete genome sequence of phage LPST10 may reveal insights into host restriction and bacteriolytic mechanisms, which will provide a basis for rational genetic modification to enhance therapeutic applications.

DNA from LPST10 was extracted and purified according to reference 6. The genome was sequenced with the Illumina HiSeq platform, which produced 60,446 raw paired-end reads of 151 bp. A total of 54,614 reads were assembled into a complete genome using Newbler version 2.8 software. The genomic length of phage LPST10 was 47,657 bp, with a GC content of 45.53%. The complete genome sequence of phage LPST10 has a high similarity to those of *Salmonella* phages 64795_sal3 (GenBank accession no. KX017520), IME207 (GenBank accession no. KX523699), and E1 (GenBank accession no. AM491472.1), with coverages of 72%, 59%, and 53%, respectively. The annotation results from the RAST server (7) predicted that phage LPST10 contained 87 putative open reading frames (ORFs). Only 29 ORFs were predicted to encode proteins with known functions. The products of the 21 ORFs belong to the phage structure and packaging module (containing the neck whisker protein, tape measure protein, phage tail protein, tail fiber protein, tail assembly protein, head decoration protein, and coat protein); the replication/transcription module (containing transcriptional regulators, homing endonuclease, single-stranded DNA-binding protein, recombinase, exonuclease, DNA primase/helicase, terminase large subunit, and DNA polymerase III beta subunit); and the host lysis module (containing the holin protein and lysozyme). Remarkably, phage LPST10 has two different endolysins. No putative virulence factors were identified according to Virulence Factor Database (VFDB [see http://www.mgc.ac.cn/VFs/main.htm]). No antibiotic resistance genes were screened using the Antibiotic Resistance Gene Database (8). The results of this study suggest a possible use of phage LPST10 as a prophylactic agent for the control of *Salmonella* species.

### Accession number(s).

The complete genome sequence of *Salmonella* phage LPST10 has been deposited in GenBank under the accession number KY860935.
